# Voice of a woman: influence of interaction partner characteristics on cycle dependent vocal changes in women

**DOI:** 10.3389/fpsyg.2024.1401158

**Published:** 2024-12-13

**Authors:** Janek S. Lobmaier, Wilhelm K. Klatt, Stefan R. Schweinberger

**Affiliations:** ^1^Department of Clinical Psychology and Psychotherapy, University of Zurich, Zurich, Switzerland; ^2^Sigma-Zentrum für Akutmedizin, Fachkrankenhaus für Psychiatrie, Psychotherapie, Psychosomatische Medizin, Bad Säckingen, Germany; ^3^Department for General Psychology and Cognitive Neuroscience, Institute of Psychology, Friedrich Schiller University, Jena, Germany; ^4^Swiss Center for Affective Sciences, University of Geneva, Geneva, Switzerland

**Keywords:** menstrual cycle, social mimicry, accommodation, attractiveness, phonetics

## Abstract

**Introduction:**

Research has shown that women’s vocal characteristics change during the menstrual cycle. Further, evidence suggests that individuals alter their voices depending on the context, such as when speaking to a highly attractive person, or a person with a different social status. The present study aimed at investigating the degree to which women’s voices change depending on the vocal characteristics of the interaction partner, and how any such changes are modulated by the woman’s current menstrual cycle phase.

**Methods:**

Forty-two naturally cycling women were recorded once during the late follicular phase (high fertility) and once during the luteal phase (low fertility) while reproducing utterances of men and women who were previously assessed to have either attractive or unattractive voices.

**Results:**

Phonetic analyses revealed that women’s voices in response to speakers changed depending on their menstrual cycle phase (F0 variation, maximum F0, Centre of gravity) and depending on the stimulus speaker’s vocal attractiveness (HNR, Formants 1–3, Centre of gravity), and sex (Formant 2). Also, the vocal characteristics differed when reproducing spoken sentences of the stimulus speakers compared to when they read out written sentences (minimum F0, Formants 2–4).

**Discussion:**

These results provide further evidence that women alter their voice depending on the vocal characteristics of the interaction partner and that these changes are modulated by the menstrual cycle phase. Specifically, the present findings suggest that cyclic shifts on women’s voices may occur only in social contexts (i.e., when a putative interaction partner is involved).

## Introduction

Social interactions are an integral part of our daily lives. In the vast tapestry of human social interactions, the voice serves as a powerful medium to convey not only factual information, but also rich dynamic information about the speaker’s identity, emotions, and intentions (Schweinberger et al., 2014). From an evolutionary perspective, the voice may be understood as an “honest signal,” a concept rooted in the theory that certain traits and behaviors have evolved to convey truthful information to others about the individual’s fitness, health, or reproductive status. The human voice, particularly in women, may carry indicators of reproductive viability or general health. The pitch and tonal quality of a woman’s voice can signal her age and hormonal status, which are closely linked to fertility. In fact, research suggests that women’s voices sound most attractive during the high-fertility phase of the menstrual cycle (Pipitone and Gallup, 2008). Meanwhile, there is also evidence that people change their voices depending on the context, for example when speaking to a highly attractive person or a person with a different social status (e.g., Fraccaro et al., 2011; Hughes et al., 2010; Zraick et al., 2006). The aim of the present study was to investigate the extent to which women’s voices change depending on the vocal characteristics of the interaction partner, and whether such changes are modulated by the current phase of the woman’s menstrual cycle.

Hearing a person’s voice allows a listener to form impressions about the speaker’s personality. For example, men and women with a lower voice pitch are perceived as more competent and trustworthy than those with higher-pitched voices. Conversely, women with a higher voice pitch are judged as having a warmer personality than women with lower-pitched voices (Oleszkiewicz et al., 2017). In return, a speaker’s voice can be adapted, according to the circumstances in which a conversation takes place. For instance, women have been shown to speak with higher voice pitch, larger pitch range, expanded intonation contours, and slower speech rate when speaking to infants (i.e., “motherese”), compared to when speaking to adults (Fernald and Simon, 1984; Grieser and Kuhl, 1988), and this phenomenon has been shown to be universal in western and traditional cultures (Broesch and Bryant, 2015). Voice pitch is also affected by changes in tension (Titze, 1989), intonation, stress, and loudness of speech (Raphael et al., 2011), and women have been reported to speak in a lower or higher voice pitch when speaking to a superior or a subordinate, respectively (Zraick et al., 2006).

Voice pitch is typically measured by the mean fundamental frequency (mean F0), depicting the rate of vocal fold vibration. The standard deviation of the fundamental frequency (F0 SD) represents the variability in perceived voice pitch (i.e., intonation). Low values in F0 SD are perceived as a monotonous voice, higher values are found in melodious voices. Other measures often used to objectively measure voice characteristics include minimum fundamental frequency (F0_min_) and maximum fundamental frequency (F0_max_), Centre of gravity, Formants 1 to 4 (F1–F4), harmonics-to-noise ratio (HNR), Jitter, Shimmer, and variation in intensity (Intensity SD). F0_min_ and F0_max_ represent the upper and lower limits of the pitch range. Centre of gravity is the frequency which divides the voice spectrum into two halves, so a higher Centre of gravity means that a voice has more high-frequency energy in its spectrum. F1 to F4 are frequency ranges that are intensified within the spectrum. HNR is the ratio of harmonic to non-harmonic components within the voice spectrum and reflects breathiness of the voice. Jitter is a local variation in frequency, Shimmer is a local variation in amplitude; both Jitter and Shimmer are caused by irregular vocal fold vibration and are perceived as roughness in a speaker’s voice. Finally, Intensity SD indicates the variability in perceived loudness.

People seem to alter their voice when speaking to interaction partners they find attractive. Fraccaro et al. (2011) found that women speak at a higher pitch (higher mean F0) to men to whom they are attracted, whereas Hughes et al. (2010) reported that both women and men lower their voice pitch (mean F0) when speaking to an attractive opposite-sex target. When speaking to attractive women, both men and women appear to show a greater voice pitch variability (higher F0 SD; Leongómez et al., 2014). Farley et al. (2013) provided evidence suggesting that naïve listeners were able to identify whether a person was speaking to a romantic partner or a friend. Women used a deeper voice (lower mean F0) when talking to their romantic partners than to their friends. In contrast, men used a higher voice pitch when addressing their romantic partners. As a limitation, this study compared voice samples directed to opposite-sex partners and voice samples directed to same-sex friends, conflating sex of the interaction partner with intimacy. Nevertheless, these studies together suggest that women change their voices depending on the sex and attractiveness of the interaction partner. However, none of these studies controlled for a potential influence of the menstrual cycle.

Regarding attractiveness of voices, there is evidence that attractiveness increases with “averageness” of a voice – an effect that is potentially enhanced by larger harmonics-to-noise ratio in attractive voices (Bruckert et al., 2010; Zaske et al., 2020a). Several studies suggest that women’s voices, and voice attractiveness in particular, change during the menstrual cycle. Pipitone and Gallup (2008) found that the attractiveness of naturally cycling women’s voices increases with their conception probability. In a follow-up study, Shoup-Knox and Pipitone (2015) demonstrated that voices during high fertility period are not only rated as more attractive than low fertility voices but also lead to a higher galvanic skin response in the listeners, suggesting an increased arousal at a physiological level. Shoup-Knox et al. (2019) analysed Pipitone and Gallup’s (2008) recordings phonetically, finding a significantly lower Shimmer in high fertility compared to low fertility recordings of naturally cycling women. Bryant and Haselton (2009) found that women’s voice pitch (mean F0) was increased during high compared to low fertility when speaking the sentence “Hi, I’m a student at UCLA.” At first sight, these findings suggest that a higher pitch in women’s voices might convey a cue to fertility. Karthikeyan and Locke (2015) replicated the finding that highly fertile women’s voices are rated as more attractive, but they found that the women in their sample actually spoke in a *lower* voice pitch compared to when not fertile. A further study recorded women’s voices on a daily basis throughout their cycle (Fischer et al., 2011), finding that mean voice pitch and variation in voice pitch increase prior to ovulation and show a distinct drop on the day of ovulation.

Consistently, men rated the voices to be more attractive during the pre-ovulatory period than on the day of ovulation itself (Fischer et al., 2011). Banai (2017) compared the voices of naturally cycling women and women using hormonal contraceptives during menstruation, the late follicular and the luteal phase. She found naturally cycling women to have a higher *minimum* voice pitch (F0_min_) in the late follicular phase and a lower voice intensity in the luteal phase, each compared to the other phases. In hormonal contraceptive users, no voice changes across the cycle were detected. In contrast to Fischer et al. (2011), Banai (2017) did not find a significant effect in *mean* voice pitch (mean F0). This indicates that mean F0 alone does not seem to be a reliable cue to fertility. Similar to Banai (2017), La and Polo (2020) compared the voices of naturally cycling women with those taking hormonal contraceptives at three different times of the cycle, but in a double-blind design. In naturally cycling women, they found a significantly lower mean F0, F0 SD, and maximum F0 during menstruation, compared to the follicular and luteal phase. In women using hormonal contraceptives there was no cycle-dependent difference in these measures.

However, overall, and despite considerable variability in study designs and findings reported above, a sizeable number of published findings indicate the existence of voice changes depending on the menstrual cycle.

A common explanation for cycle-dependent voice changes is based on the fact that levels of women’s reproductive hormones vary during the menstrual cycle, with a surge of estradiol in the late-follicular phase and high levels of progesterone in the luteal phase. Regarding the vocal apparatus, estradiol promotes cell differentiation and mucosa secretion, while progesterone enhances the acidity and viscosity of the mucus for water retention, resulting in increased mass of the vocal folds, which in turn promotes lower-frequency vibration (Abitbol et al., 1999; Karthikeyan and Locke, 2015). In addition, there is evidence for specific sex hormone receptors within the vocal fold mucosa (Schneider et al., 2007; but see Nacci et al., 2011). Together, these studies suggest that a woman’s vocal apparatus may change under the influence of cycle dependent hormone concentration, leading to perceivable changes in her voice.

It should be noted that not all studies found an effect of the menstrual cycle on women’s voices. For example, Barnes and Latman (2011) found no significant voice changes across the cycle and no differences between naturally cycling women and those taking hormonal contraceptives in mean F0, jitter, shimmer, relative average perturbation, peak-to-peak amplitude variation, HNR, degree of voice breaks, and number of voice breaks. Likewise, Meurer et al. (2009), Raj et al. (2010), Celik et al. (2013), and Plexico et al. (2020) found no evidence for cycle-dependent voice changes.

An alternative explanation for cycle effects on women’s voices – which might also explain conflicting results – refers to psychological changes which may lead to increased mating motivation when currently fertile (Haselton and Gildersleeve, 2016). Karthikeyan and Locke (2015) suggest that only when provided with a mating context, women may be motivated to speak and behave more attractively, showing subtle vocal behaviors that are phase-specific (see also Klatt et al., 2020). However, even within a mating context, Pavela Banai et al. (2022) did not detect cycle-related changes in mean F0 and F0 SD in naturally cycling women who were leaving voice messages directed to masculinized and feminized pictures of men and women. Given these inconsistent results, Pavela Banai et al. (2022) name several methodological issues in earlier studies on cycle-dependent voice changes. First, different cycle-tracking methods have been used (counting method, body temperature, assessment of hormone levels). Second, many studies focused on mean F0 only, disregarding other voice parameters. Third, a variety of vocal stimuli have been analysed (vowels, numbers, sentences, free speech) which differ in experimental control and ecological validity. For example, women’s mean F0 has been shown to differ significantly depending on whether they were counting, producing vowels, reading out, or speaking spontaneously (Zraick et al., 2000).

In addition to these issues, one could argue that when a mating context is created, it makes a difference whether or not you are leaving a voice message for a target with an attractive face (Hughes et al., 2010; Pavela Banai et al., 2022) or an attractive voice (e. g., Karthikeyan and Locke, 2015). Furthermore, we want to point out that for phonetic analyses, previous studies used different software applications and different frequency ranges when analysing voice recordings for mean F0, F0 SD, F0_min_, and F0_max_. It is reasonable that phonetic analyses will produce different results when the predefined search area for F0 parameters is different (see Table 1 for an overview of different studies and the respective F0 definitions). Also, despite the fact that mean F0 may be the easiest voice parameter to quantify, researchers interested in social mimicry of voices are well advised to also quantify other relevant vocal parameters.

**Table 1:** Overview of frequency ranges used in phonetic analyses in previous studies.

Study	Software	Frequency range analysed for F0
Banai (2017)	Praat	100–500 Hz
Pavela Banai et al. (2022)	Praat	100–500 Hz
Barnes and Latman (2011)	N/A	N/A
Bryant and Haselton (2009)	Praat	100–600 Hz
Celik et al. (2013)	Praat	N/A
Farley et al. (2013)	Praat	N/A*
Feinberg et al. (2006)	Praat	100–600 Hz
Fischer et al. (2011)	Kurt Hammerschmidt’s custom acoustic analysis tool	Automatic**
Fraccaro et al., 2011, referring to Feinberg et al. (2008), referring to Feinberg et al. (2005)	Praat	100–600 Hz
Hughes et al. (2010)	Praat	75–600 Hz***
Karthikeyan and Locke (2015)	Multi-speech software	75–300 Hz
La and Polo (2020)	Electrolaryngographic recording, sopran software by svante granqvist	N/A
Leongómez et al. (2014)	Praat	100–500 Hz
Meurer et al. (2009)	Motor speech profile program	N/A
Plexico et al. (2020)	Computerized speech laboratory; analysis of dysphonia in speech and voice program	E-Mail 08.10.23, no answer
Raj et al. (2010)	Vaughmi software	N/A
Shoup-Knox et al. (2019)	Praat	E-Mail 08.10.23, no answer

*Personal communication via e-mail, 16.09.2023. **Personal communication via e-mail, 15.09.2023. ***Personal communication via e-mail, 15.09.2023.

Given that women’s voices can change depending on their current menstrual cycle phase, characteristics of the interaction partner and speech context, we aimed at further scrutinizing potential internal and external factors that may have an effect on women’s voices. Specifically, as an internal factor of the participants, we tested whether the menstrual cycle phase affects women’s voices. In addition, as an external factor, we manipulated the interaction partner of the women (operationalized here as the stimulus speaker). This interaction partner could either be a man or woman, with an attractive or unattractive voice. In contrast to studies in which conception probability was calculated using counting methods (Pipitone and Gallup, 2008; Puts et al., 2013), we verified the menstrual cycle phases by hormone tests based on urine (luteinising hormone LH) and saliva (estradiol, progesterone). We hypothesized that women alter their voices depending on sex and attractiveness of the stimulus speaker’s voice, and that these changes are modulated by the current menstrual cycle phase of the women. An effect of menstrual cycle was thought to occur more pronounced in response to men’s voices than to women’s voices, and more pronounced in response to attractive voices than to unattractive voices. Voice recordings were analysed phonetically for a large range of parameters that included mean F0, F0_min_, F0_max_, F0 SD, HNR, Jitter, Shimmer, Formants 1 to 4, Centre of gravity, and Intensity SD.

## Materials and methods

### Participants

Eighty-three women were initially recruited for the present study. Participants were recruited from the subject pool of the university, via flyers and leaflets, the institutional website, free internet advertisements, and word-of-mouth recommendation. All of them provided written informed consent to participate and the study was approved by the local ethics committee (approval number: 2012-8-167070) and participants were treated in accordance with the Code of Ethics of the World Medical Association (Declaration of Helsinki). They were compensated either with course credits or 50 CHF (approximately 60 US$). The final sample consisted of 42 women (see below).

### Stimuli

Recordings of stimulus speakers (spoken sentences) were taken from the Jena Speaker Set (JESS; Zaske et al., 2020b) which at the time consisted of 64 speakers (half of them women) who were recorded while speaking a series of neutral sentences. All sentences consisted of exactly five words and had the same syntactic structure (e.g., “Der Fahrer lenkt den Wagen”/“The driver steers the car,” see Supplementary Table S1 for the complete list of sentences used).

In order to identify the most attractive and most unattractive voices of the 64 stimulus speakers, an attractiveness rating was performed with an independent sample of participants using PsychoPy software (Peirce, 2007). Twenty-four listeners (half of them women, ranging in age between 20 and 39 years, *M* = 25.0, *SD* = 4.6) who were recruited via flyers and word-of-mouth recommendation were received individually in a quiet room. After giving informed consent, they were asked to take place in front of a laptop and to attach Sennheiser HD 439 headphones. Listeners were told that they would be hearing spoken sentences via headphones and that they were asked to respond to the question “How attractive do you find this voice?” on a 7-point likert scale that was showed on the screen (labelled “very attractive” and “very unattractive” at each end). Instructions were given verbally and onscreen. Listeners first completed three practice trials with sentences and stimulus speakers which were not used afterwards to get used to the task and to adjust the volume of the headphones. Voice recordings of the 64 stimulus speakers uttering the same two neutral sentences (“The train passes the town,” “The lemonade quenches the thirst”) were presented and rated for attractiveness separately in randomised order, resulting in 128 trials. The experiment took about 10 min to complete. Rank analyses identified the eight most attractive and eight most unattractive female and male speakers, respectively, resulting in 32 different stimulus speakers which were then used in the present study.

### Procedure

Initially, all interested women were asked to complete an online survey with questions regarding age, smoking habits, mother tongue, reading disabilities, hearing problems, sexual orientation, relationship status, use of hormonal contraception, pregnancy, onset of last menstruation, regularity and length of menstrual cycle. To take part in this study, participants had to meet following inclusion criteria: No use of hormonal contraceptives (contraceptive pill, morning-after pill, hormonal contraceptive coil, contraceptive implants); no pregnancy, and no breastfeeding within the last three months; regular menstrual cycle (23–35 days in length); heterosexual orientation; German mother tongue; no dyslexia; no hearing problems; no chronic smoking (more than 20 cigarettes a week). All reported to be healthy (no mental and/or physical diseases) and not to have a hoarse voice, cough, or nasal congestion on the days of recording. Women who met the above-mentioned inclusion criteria were contacted by phone by a female research assistant who gave them detailed information about the procedure.

To determine time of highest fertility, participants completed a series of urine tests measuring a metabolite of luteinising hormone (LH) using one-step urine LH tests with a reported sensitivity of 10 mIU/ml (David One Step Ovulation Tests, Runbio Biotech, China, http://www.runbio-bio.com). Women were instructed to perform urine tests twice a day (morning and evening) starting three days before the date of predicted peak fertility (based on the average cycle length of each individual woman using forward and backward counting method). After a positive test result, participants continued performing LH tests until the results became negative for two consecutive days. Participants photographed each test using their smartphones and sent the picture to the research assistant, who verified whether the test was positive or not.

The women were either scheduled to be tested approximately two days before the calculated day of peak fertility and again seven days after a positive LH test result (late follicular–luteal menstrual cycle condition) or they were scheduled seven days after the LH surge (luteal–late follicular menstrual cycle condition). Participants of the luteal–late follicular menstrual cycle condition performed LH tests again in the following cycle and were scheduled to be tested two days before the calculated day of peak fertility. Thus, LH tests were used to determine peak fertility and to verify that the cycle was ovulatory. Late follicular recording sessions took place between 4 days before and 24 h after the LH surge, luteal phase recording sessions took place 6 to 13 days after the LH surge (see Table 2 for an overview of the time of recording relative to the LH surge). Order of recording sessions was counterbalanced across participants: half of the women completed the first session at high fertility and their second session in the luteal phase (late follicular–luteal menstrual cycle condition), and the other half were tested first during the luteal phase and then during high fertility (luteal–late follicular menstrual cycle condition).

**Table 2:** Time of recording sessions relative to peak fertility as indicated by LH surge.

Days relative to LH surge	Participants in late follicular session	Days relative to LH surge	Participants in luteal session
– 4	2	+ 6	5
– 3	3	+ 7	8
– 2	4	+ 8	12
– 1	9	+ 9	10
0	13	+ 10	2
+ 1	11	+ 11	3
		+ 12	1
		+ 13	1

In order to assess phase-specific hormone levels, participants provided saliva samples from which estradiol, progesterone, testosterone, and cortisol levels were determined. Participants were instructed to refrain from eating and drinking anything but water for at least 30 min prior to saliva collection. Samples were collected by passive drool using a commercially available sampling device (SaliCaps, IBL International, Hamburg, Germany). The saliva samples were stored at −28°C and were later analysed by an independent laboratory (Dresden Lab Service GmbH, Dresden, Germany) using liquid chromatography with coupled tandem mass spectrometry (LC–MS/MS). LC–MS/MS has become the method of choice for steroid analysis because of its high sensitivity, better reproducibility, greater specificity, and ability to analyse multiple steroids simultaneously (see Gao et al., 2015 for methodological details on LC–MS/MS). Both recording sessions took place between 8 and 11 AM in order to control for circadian variability of hormone levels (Dabbs and Delarue, 1991; Wust et al., 2000).

Women’s voices were recorded in a soundproof recording booth under standardised conditions. A Beyerdynamic MC 930 condenser microphone with a popkiller and 48 V phantom power was placed about 20 centimetres away from the participant’s mouth and connected to a Zoom H4n digital audio recorder (uncompressed WAV, 48 kHz, 16-bit sampling rate). Before starting the experiment proper, participants were asked to read out a short newspaper article about voice research presented on a computer screen to warm up their voices and to adjust the recording level to −12 to −6 dB. Each woman absolved two recording sessions (one in the late follicular and one in the luteal phase), and each recording session consisted of two blocks. In the first block the woman read aloud presented written sentences; in the second block, the task was to reproduce spoken sentences verbally which were presented via headphones. Written and spoken sentences were identical in content. At the beginning of the first block, the instruction to read out presented written sentences in a natural manner was given verbally and onscreen. Twenty-four sentences of affectively neutral content (see Supplementary Table S1) were presented. Sentences were displayed on a laptop screen in 25 pt. Arial using PsychoPy software (Peirce, 2007). The screen was placed in front of the participant at a distance of approximately 50 centimetres. Sentences were presented consecutively in randomised order for five seconds each. After three practice trials with sentences which were not used subsequently, the experimenter left the booth and the participant started the first block of the recording session (written sentences). The participant indicated by knocking on the booth’s door when the block was finished. The experimenter returned, asked the participant to attach Sennheiser HD 439 headphones and prepared the second block of the recording session (spoken sentences). Participants were told that they would be hearing spoken sentences via headphones and that they were to reproduce these sentences in a natural manner. Instructions were given verbally and onscreen. Sentences were presented separately in randomised order. Participants first completed three practice trials with sentences and stimulus speakers which were not used in the experiment proper. Subsequently, they were given the opportunity to adjust the volume of the headphones. After that, the experimenter left the booth and the participant started the second block (spoken sentences). Again, the participant indicated by knocking on the booth’s door when the block was finished. Both recording sessions (late follicular and luteal phase) consisted of these two blocks, following the same procedure except that participants were fully debriefed after the second recording session. Each recording session took about 45 min to complete.

Of the 83 women who initially entered the study, some had to be excluded because their recordings were unusable due to misspeaking (*N* = 12), because they had anovulatory cycles during the recording period (i.e., no LH surge, *N* = 8), did not conduct the required LH tests during the peri-ovulatory period (*N* = 3), were tested in the wrong cycle phase (too early/too late as revealed by LH tests, *N* = 8), or dropped out due to personal reasons (*N* = 10). Thus, the final sample consisted of 42 women between 19 and 35 years of age (*M* = 22.6, *SD* = 3.4). From these, a total of 2,016 uncompressed WAV voice samples (42 participants × 2 menstrual cycle phases × 24 sentences) were cut from raw recordings using Audacity^®^ software (Audacity Team, 2023).

### Variables and statistical analyses

Praat software version 6.3.16 (Boersma and Weenink, 2023) was used to analyse the voice samples for the following phonetic parameters: Mean F0, F0 SD, F0_min_, F0_max_, Centre of gravity, Formants 1 to 4, HNR, Jitter, Shimmer, and Intensity SD. For analyses of mean F0, F0 SD, F0_min_, and F0_max_, the frequency range was set to 100–500 Hz and for formant analyses, the maximum frequency was set to 5,500 Hz, as suggested by Boersma and Weenink (2023) for female voices. Apart from that, default settings were used.

#### Control condition: reading written sentences aloud

In the control condition (where participants read the sentences out loud from the computer screen), for each woman, values of individual vocal parameters were averaged over the 24 sentences, separately for both menstrual cycle phases for further analyses. We then calculated one-factor (participant’s menstrual cycle phase: Late follicular vs. luteal phase) repeated measures ANOVA’s, separately for each of the vocal parameters using Bonferroni correction.

We also ran Pearson correlations between the hormone levels and the vocal parameters, separately for the late follicular and luteal phase. We present these correlations in the Supplementary material.

#### Experimental condition: reproduction of spoken sentences

In the Experimental condition (where participants reproduced the sentences after hearing them being spoken), for each woman, values of individual vocal parameters were averaged over the individual sentences, separately for menstrual cycle phase of the woman, stimulus speaker’s vocal attractiveness, and stimulus speaker’s sex, for further analyses. We then calculated 2 (participant’s menstrual cycle phase: Late follicular vs. luteal phase) × 2 (stimulus speaker’s vocal attractiveness: Attractive vs. unattractive) × 2 (stimulus speaker’s sex: Female vs. male) repeated measures ANOVA separately for each of the vocal parameters. The Huynh-Feldt epsilon correction for heterogeneity of covariances (Huynh and Feldt, 1976) was used when sphericity could not be assumed. For post-hoc pairwise comparisons we used the Bonferroni correction. To correct for multiple testing, we used the Holm-Bonferroni method. We report the corrected alongside the uncorrected *p*-values.

We also ran Pearson correlations between the hormone levels and the vocal parameters, separately for the late follicular and luteal phase. We present these correlations in the Supplementary material.

#### Phonetic analysis of the stimulus speakers’ voices

To examine whether the effects in women’s voices in reaction to the stimulus speakers’ voice characteristics may be driven by social mimicry or accommodation, also the stimulus speakers’ voices were analysed phonetically for the same parameters as the participants using Praat software version 6.3.16 (Boersma and Weenink, 2023). Female and male stimulus speakers’ voices were analysed separately because of sex-specific pre-adjustments in the phonetic software (Leongómez et al., 2014). For analyses of mean F0, F0 SD, F0_min_, and F0_max_ of the female stimulus speakers, frequency range was set to 100–500 Hz; for formant analyses, the maximum frequency was set to 5,500 Hz (same as for analyses performed with the participants’ voices). For analyses of mean F0, F0 SD, F0_min_, and F0_max_ of the male stimulus speakers, frequency range was set to 75–300 Hz;for formant analyses, the maximum frequency was set to 5,000 Hz, as suggested by Boersma and Weenink (2023). Apart from that, default settings were used.

## Results

### Control condition: reading written sentences aloud

The one-factor (participant’s menstrual cycle phase: Late follicular vs. luteal phase) repeated measures ANOVAs indicated no effect of menstrual cycle phase on any of the phonetic measures (all *p*s > 0.30). ANOVA results and descriptive statistics are provided in Table 3.

**Table 3:** Control condition (reading aloud written sentences): phonetic parameters depending on participants’ menstrual cycle phase.

	Menstrual cycle phase				ANOVA		
	Late follicular		Luteal		(follicular vs. luteal)		
	*M*	*SD*	*M*	*SD*	*F*	*p*	*η_p_^2^*
F0	213.25 Hz	16.75	213.39 Hz	17.46	0.011	0.92	0.000
F0 SD	44.35 Hz	8.85	44.67 Hz	9.76	0.067	0.80	0.002
F0_min_	147.99 Hz	19.31	148.32 Hz	17.94	0.021	0.89	0.001
F0_max_	386.88 Hz	40.25	389.12 Hz	39.89	0.127	0.72	0.003
Centre of gravity	684.99 Hz	254.10	668.83 Hz	193.61	0.936	0.34	0.022
F1	674.71 Hz	42.28	678.43 Hz	36.78	0.377	0.54	0.009
F2	1842.96 Hz	53.84	1842.06 Hz	56.57	0.041	0.84	0.001
F3	2863.71 Hz	59.82	2860.69 Hz	63.29	0.288	0.60	0.007
F4	3925.51 Hz	63.81	3923.96 Hz	65.48	0.052	0.82	0.001
HNR	13.31 dB	2.21	13.17 dB	2.16	1.027	0.32	0.024
Jitter	0.03%	0.01	0.03%	0.01	0.511	0.48	0.012
Shimmer	0.09%	0.01	0.09%	0.01	0.000	1.00	0.000
Intensity SD	11.14 dB	1.37	11.25 dB	1.20	0.473	0.50	0.011

### Experimental condition: reproduction of spoken sentences

The 2 (participant’s menstrual cycle phase: Late follicular vs. luteal phase) × 2 (stimulus speaker’s vocal attractiveness: Attractive vs. unattractive) × 2 (stimulus speaker’s sex: Female vs. male) repeated measures ANOVA was calculated on each of the vocal parameters. The results of the 2 (late follicular vs. luteal phase) × 2 (attractive vs. unattractive) × 2 (stimulus speaker’s sex) repeated measures ANOVAs are shown in Table 4, descriptive statistics are given in Table 5. Menstrual cycle phase of the participants had a significant main effect on F0 SD, F0_max_, and Centre of gravity. When recorded in the late follicular phase, women spoke with lower F0 SD, lower F0_max_, and higher Centre of gravity compared to the luteal phase. When correcting for multiple testing, menstrual cycle phase had an effect only on F0_max_. Stimulus speaker’s voice attractiveness showed a significant effect on five phonetic measures. HNR was lower in response to stimulus speakers with attractive voices than in response to speakers with less attractive voices, implying that participants used a breathier and hoarser voice when reacting to attractive voices than when reacting to unattractive voices. Formants 1 to 3, reflecting frequencies that are intensified relative to the rest of the vocal spectrum, were significantly higher in frequency when responding to attractive stimulus speakers compared to unattractive stimulus speakers. Additionally, women showed a higher frequency in Centre of gravity in response to attractive stimulus speakers compared to unattractive stimulus speakers. When correcting for multiple testing, the effect remained significant only for Center of Gravity and F1. The sex of the stimulus speaker had a significant effect only on Formant 2, which was higher in frequency when women responded to a female stimulus speaker, compared to when responding to a male stimulus speaker. This effect was also significant after correcting for multiple testing. No significant main effects were observed in mean F0, F0_min_, Jitter, Shimmer, Formant 4 and Intensity SD. No interaction reached statistical significance (all *p*s ≥ 0.05; see Supplementary Table S2).

**Table 4:** Experimental condition (reproduction of spoken sentences), inferential statistics: phonetic parameters depending on menstrual cycle phase of the participants, voice attractiveness of the stimulus speaker, and sex of the stimulus speaker.

	Participant’s menstrual cycle phase (follicular vs. luteal)				Stimulus speaker’s voice attractiveness (attractive vs. unattractive)				Stimulus speaker’s sex (female vs. male)			
	F	*p*	*p* (corr)	*η_p_^2^*	F	*p*	*p* (corr)	*η_p_^2^*	F	*p*	*p* (corr)	*η_p_^2^*
F0	0.510	0.48	>0.999	0.012	0.472	0.50	>0.999	0.011	0.689	0.41	>0.999	0.017
F0 SD	9.008	**0.005**	0.06	0.180	2.136	0.15	>0.999	0.050	0.468	0.50	>0.999	‘011
F0_min_	3.46	0.07	0.7	0.078	0.467	0.50	>0.999	0.011	2.584	0.116	>0.999	0.059
F0_max_	15.379	**0.001**	**0.013**	0.273	1.570	0.22	>0.999	0.037	0.911	0.35	>0.999	0.022
CoG	5.128	**0.029**	0.319	0.111	13.601	**0.001**	**0.013**	0.249	0.079	0.78	>0.999	0.002
F1	0.215	0.65	>0.999	0.005	17.907	**<0.001**	**0.013**	0.304	2.611	0.11	>0.999	0.060
F2	0.218	0.353	>0.999	0.009	8.922	**0.005**	0.055	0.179	11.436	**0.002**	**0.026**	0.218
F3	0.548	0.46	>0.999	0.013	7.364	**0.01**	0.09	0.152	0.171	0.68	>0.999	0.004
F4	0.546	0.50	>0.999	0.011	0.332	0.57	>0.999	0.008	1.703	0.20	>0.999	0.040
HNR	1.822	0.18	>0.999	0.043	8.885	**0.005**	0.055	0.178	0.260	0.61	>0.999	0.006
Jitter	0.093	0.76	>0.999	0.002	0.882	0.35	>0.999	0.021	1.277	0.27	>0.999	0.030
Shimmer	0.177	0.77	>0.999	0.004	0.669	0.42	>0.999	0.016	2.196	0.15	>0.999	0.051
Intens SD	0.412	0.53	>0.999	0.010	0.115	0.74	>0.999	0.003	0.592	0.47	>0.999	0.013

*p* (corr): *p*-value corrected for multiple testing using the Holm-Bonferroni Method. Bold values indicate significant effects.

**Table 5:** Experimental condition (reproduction of spoken sentences), descriptive statistics: phonetic parameters depending on menstrual cycle phase of the participants, attractiveness of the stimulus speaker, and sex of the stimulus speaker.

	Participant’s menstrual cycle phase		Stimulus speaker’s voice attractiveness		Stimulus speaker’s sex	
		*M (SD)*		*M (SD)*		*M (SD)*
F0 [Hz]	Fol	211.94 (16.98)	Attr	212.18 (17.10)	Female	212.41 (17.09)
	Lut	212.63 (18.01)	Unattr	212.39 (17.41)	Male	212.17 (17.41)
F0 SD [Hz]	Fol	44.48 (6.93)	Attr	45.84 (7.84)	Female	45.65 (7.56)
	Lut	46.55 (8.53)	Unattr	45.19 (7.32)	Male	45.37 (7.55)
F0_min_ [Hz]	Fol	139.14 (18.24)	Attr	140.62 (18.01)	Female	140.21 (17.62)
	Lut	142.61 (16.69)	Unattr	141.13 (18.30)	Male	141.54 (18.74)
F0_max_ [Hz]	Fol	385.81 (33.24)	Attr	395.37 (36.57)	Female	394.83 (36.17)
	Lut	401.59 (40.51)	Unattr	392.04 (34.88)	Male	392.58 (34.85)
Centre of gravity [Hz]	Fol	710.03 (286.57)	Attr	692.90 (249.08)	Female	687.96 (242.36)
	Lut	664.89 (214.70)	Unattr	682.03 (240.85)	Male	686.96 (247.79)
F1 [Hz]	Fol	681.71 (36.56)	Attr	682.08 (32.00)	Female	681.08 (31.96)
	Lut	679.30 (36.12)	Unattr	678.94 (32.60)	Male	679.93 (32.61)
F2 [Hz]	Fol	1855.07 (51.74)	Attr	1855.29 (47.79)	Female	1855.56 (47.79)
	Lut	1853.03 (46.38)	Unattr	1852.81 (48.09)	Male	1852.55 (48.11)
F3 [Hz]	Fol	2872.12 (62.45)	Attr	2871.26 (58.27)	Female	2870.24 (57.69)
	Lut	2867.96 (58.02)	Unattr	2868.81 (56.78)	Male	2869.84 (57.39)
F4 [Hz]	Fol	3941.56 (52.81)	Attr	3939.70 (48.96)	Female	3940.12 (50.67)
	Lut	3937.21 (55.45)	Unattr	3939.06 (50.89)	Male	3938.64 (49.21)
HNR [dB]	Fol	13.00 (1.72)	Attr	13.05 (1.84)	Female	13.11 (1.77)
	Lut	13.20 (2.02)	Unattr	13.15 (1.81)	Male	13.09 (1.87)
Jitter [%]	Fol	0.03 (0.01)	Attr	0.03 (0.01)	Female	0.03 (0.01)
	Lut	0.03 (0.01)	Unattr	0.03 (0.01)	Male	0.03 (0.01)
Shimmer [%]	Fol	0.09 (0.01)	Attr	0.09 (0.01)	Female	0.09 (0.01)
	Lut	0.09 (0.01)	Unattr	0.09 (0.01)	Male	0.09 (0.01)
Intensity SD [dB]	Fol	11.04 (1.28)	Attr	10.99 (1.08)	Female	10.98 (1.09)
	Lut	10.94 (1.11)	Unattr	10.98 (1.10)	Male	10.99 (1.08)

“fol” = participant in the late follicular phase, “lut” = participant in the luteal phase, “attr” = stimulus speaker with an attractive voice, “unattr” = stimulus speaker with an unattractive voice.

### Phonetic analysis of the stimulus speakers’ voices

Phonetic analyses of the stimulus speakers revealed that stimulus speakers with attractive voices of both sexes had higher formants and a higher Centre of gravity than stimulus speakers with less attractive voices (for more detailed results, see Supplementary Tables S3, S4). The differences in these parameters were reflected in women’s voices when responding to attractive versus unattractive stimulus speakers (Formants 1–3, centre of gravity).

Regarding stimulus speaker’s sex, not surprisingly, analyses showed that female stimulus speakers had higher frequencies in mean F0, F0 SD, F0_min_, F0_max_, and Formants 2–4 than male stimulus speakers, as well as higher voice quality according to higher HNR, lower Jitter, and lower Shimmer (see Supplementary Table S5). The difference in Formant 2 was reflected in women’s voices when responding to male versus female stimulus speakers.

Taken together, phonetic analyses of the stimulus speakers’ voices suggest that the shifts observed in women’s voices – depending on stimulus speaker attractiveness and sex – are at least in part explainable by social mimicry or accommodation (Chartrand and Lakin, 2013; Gregory and Webster, 1996).

### General effect of the predefined frequency range in phonetic analysis

Previous studies (e.g., Hughes et al., 2010; Karthikeyan and Locke, 2015) used different frequency ranges in phonetic analysis of their voice recordings compared to other studies (e.g., Leongómez et al., 2014). To test whether phonetic analyses produce different results depending on the search area in frequency range, we repeated the phonetic analysis with Praat software’s default frequency range (75–600 Hz instead of 100–500 Hz as suggested for female voices by Boersma and Weenink, 2023). As expected, the results of mean F0, F0 SD, F0_min_, and F0_max_ differed significantly between predefined frequency range of 75–600 Hz and 100–500 Hz. Also, HNR and Shimmer were significantly different when Praat’s default frequency range was used (see Supplementary Table S6, S7).

### Hormone assays

Hormone levels of the participants during the late follicular and luteal phase are shown in Table 6. A Kolmogorov–Smirnov test indicated that hormonal data were not normally distributed. Hence, nonparametric Wilcoxon signed-rank tests were used to compare the hormone levels between both cycle phases. These analyses revealed that, as expected, progesterone levels were significantly higher in the luteal phase than in the late follicular phase (*Z* = −3.748, *p* < 0.001). Levels of estradiol (*Z* = −1.389, *p* = 0.17), testosterone (*Z* = −0.312, *p* = 0.76), and cortisol (*Z* = −0.772, *p* = 0.44), however, did not differ between the two phases.

**Table 6:** Hormone levels in the two cycle phases of the participants.

	Estradiol (pg/ml)	Progesterone (pg/ml)	Testosterone (pg/ml)	Cortisol (nmol/l)
Late follicular (*M*, *SD*)	3.47 (1.49)	37.44 (70.94)	10.97 (4.52)	8.21 (5.51)
Luteal phase (*M*, *SD*)	4.23 (1.98)	110.36 (100.55)	11.69 (6.22)	7.02 (4.88)
Wilcoxon signed-rank	*p* = 0.17	***p* < 0.001**	*p* = 0.76	*p* = 0.44

Bold values indicate significant effects.

We found no significant correlation between hormones and vocal parameters during the late follicular phase, neither in the baseline condition (reading sentences out loud) nor in the treatment condition (repeating spoken sentences). During the luteal phase, however, estradiol levels were negatively correlated with mean F0 values and HNR, and positively correlated with jitter and shimmer. In the treatment condition, testosterone levels were negatively correlated with F1, F2, F3 and F4. Cortisol levels were negatively correlated with F0_min_ and F1. Estradiol levels were positively correlated with jitter (see correlation matrices in the Supplementary material).

## Discussion

The aim of the present study was to investigate whether and how women’s voices change during the menstrual cycle when responding to female or male speakers with attractive or unattractive voices. For this purpose, the voice of naturally cycling women was recorded during the late follicular phase and during the luteal phase while speaking sentences in response to a stimulus speaker (experimental condition) and when reading aloud the sentences from the computer screen (control condition). Based on earlier studies, we expected that the menstrual cycle of the participants would have an influence on women’s voices. We also hypothesized that women would alter their voices depending on whether they were responding to male (vs. female) stimulus speakers with attractive (vs. unattractive) voices. Phonetic analyses confirmed these predictions in part. In the experimental condition, some vocal parameters of women’s voices were indeed affected by their current menstrual cycle phase (but only F0_max_ when correcting for multiple testing), and by the vocal attractiveness (only Center of Gravity and F1 when correcting for multiple testing) and sex of the stimulus speaker (only F2, when correcting for multiple testing). We observed no interaction between cycle phase and stimulus attractiveness, suggesting that women in the fertile phase did not react specifically to attractive voices. By contrast, in the control condition, in which women merely read out sentences off a computer screen, we observed no effects of menstrual cycle on women’s voices.

In the experimental condition, where women “responded” to recordings of other speakers by repeating spoken sentences, we found some evidence for an effect of the current menstrual cycle phase on women’s vocal characteristics. In the control condition however, where the sentences had to be read aloud without any external vocal input, phonetic analyses showed no effect of participants’ current menstrual cycle phase. While this is inconsistent with some studies which found a cycle effect on women’s voices (Banai, 2017; Bryant and Haselton, 2009; Fischer et al., 2011; Tatar et al., 2015), it is in line with other studies that failed to find a menstrual cycle effect (Barnes and Latman, 2011; Celik et al., 2013; Meurer et al., 2009; Raj et al., 2010). The fact that we only found menstrual cycle effects when women responded to stimulus speakers but not when reading sentences out loud could contribute in part to understanding such inconsistencies across previous published studies and supports the notion that cycle-dependent voice changes need a social trigger to unfold.

When responding to stimulus speakers with attractive voices, women spoke with a breathier and hoarser voice, characterized by lower HNR (note that this effect just failed to reach statistical significance when correcting for multiple testing). Breathiness has been argued to be a feminine trait and is related to desirability in women (Henton and Bladon, 1985). We also observed heightened formant frequencies (in Formants 1–3, only in Formant 1 when correcting for multiple testing), and a higher Centre of gravity when the women responded to more attractive stimulus speakers compared to relatively unattractive stimulus speakers. Centre of gravity is the frequency which divides the voice spectrum into two halves, so a higher Centre of gravity when responding to attractive stimulus speakers means that women’s voices had more high-frequency energy compared to when responding to unattractive stimulus speakers. Of relevance, higher-frequency female voices have been reported to be more attractive than female voices with lower frequencies (Collins and Missing, 2003; Jones et al., 2010). Taken together, these findings suggest that women tried to make their voices sound more desirable and more attractive when speaking to stimulus speakers with attractive voices. In case of male targets, this could be a sign of increased mating motivation, whereas in case of female targets it could serve competitive needs in order to sound more attractive than a rival.

Notably, we did not observe a systematic variation in mean F0, suggesting that women did not generally speak in a higher (Fraccaro et al., 2011) or lower voice pitch (Hughes et al., 2010) when speaking to stimulus speakers with attractive voices. In contrast to Fraccaro et al. (2011) and Hughes et al. (2010), who presented the speakers with photographs of people they were allegedly speaking to, we asked our participants to respond to more or less attractive male and female voice recordings. Our finding might either relate to the inconsistency of the published findings discussed above, or alternatively suggests that the vocal channel alone may not be sufficient to evoke an effect.

With regard to sex of the stimulus speakers, women responded with a higher-frequency Formant 2 to female speakers than to male speakers. Phonetic analysis revealed that this effect might be the result of social mimicry or accommodation, as the speakers were mirroring characteristics of the stimulus speakers (Chartrand and Lakin, 2013; Gregory and Webster, 1996).

The observation that a cycle effect occurred in the experimental condition (responding to stimulus speakers, social context) but not in the control condition (reading sentences aloud, no social context) does not support the assumption that hormone-driven changes in laryngeal mucus and vocal folds are responsible for cycle dependent vocal changes (Abitbol et al., 1999). Furthermore, according to Abitbol et al. (1999), we would expect higher vocal frequencies during the follicular phase. Instead, we observed a higher F0_max_ in the luteal phase than in the follicular phase. Overall, our findings suggest that an effect of the menstrual cycle on a woman’s voice does not occur by default and as a result of hormone-driven biological inevitabilities, but instead needs a social trigger to unfold. This interpretation corresponds to the findings of Bryant and Haselton (2009) who found an effect of menstrual cycle only when women spoke a social sentence, not vowels, further suggesting that cycle-dependent voice changes may occur during social communication only. Likewise, Karthikeyan and Locke (2015) supposed that only when women are motivated to speak and behave attractively, subtle cycle-dependent voice changes may occur. Accordingly, Klatt et al. (2020) found an effect of menstrual cycle phase only if the women uttered sentences with a social content, but not when they spoke neutral sentences.

In previous studies, different software applications and different predefined frequency ranges have been used in phonetic analyses of human voices, which is problematic in terms of comparability of the results (see Table 1). By using different presettings in frequency range, we demonstrate that the chosen frequency range has an effect on phonetic raw data. We repeated phonetic analyses with the default presettings of Praat software (75–600 Hz instead of 100–500 Hz), as did for example Hughes et al. (2010). The Praat output was significantly different in mean F0, F0 SD, F0_min_, F0_max_, HNR, and Shimmer, depending on recording condition. Given these results, it is possible that the inconsistent results of previous studies on female voices partially trace back to the variety of phonetic analysis software and different frequency ranges which have been used. Voice changes during the menstrual cycle are subtle and therefore difficult to detect. In order to increase the comparability of future studies, we recommend that researchers develop unified standards for phonetic analysis. Specifically, we recommend using Praat software with the respective settings for male and female voices suggested by the Praat developers.

This study has some limitations. First, mostly Swiss-German speaking individuals with different regional dialects were asked to speak standard German during recording. Potentially this may have resulted in moderately elevated stress for the speakers, making them feeling slightly uncomfortable, and this could have affected their voices over and above any effect of their current menstrual cycle phase or the stimulus speakers’ voices. Second, reproducing the same sentences multiple times may lead to spontaneously occurring variation during speech since a speaker does not pronounce a sentence in exactly the same way every time (Fitch, 1990). This spontaneous variation might have interfered with voice changes evoked by the stimulus speaker. Thirdly, and most obviously, the participants were asked to repeat sentences spoken by stimulus speakers without any real interaction taking place. Although we opted for the present experimental design because allowing natural oral interaction between participants would have significantly confined experimental control, it is plausible that stronger social mimicry effects can be observed in a real social interaction situation. Finally, this was a complex study that demanded enormous commitment from the study participants. It is therefore understandable that some women did not take part until the end. Unfortunately, we no longer have access to the full set of data from the excluded participants, as in some cases, the ethical protocol demanded us to delete them. However, as the women left the study at different stages and for different reasons, we assume that there is no systematic reason for their quitting the study.

In the present study, we took great care in scheduling the cycle-dependent recording sessions and in standardisation of the recording procedure which has sometimes been neglected in previous studies. In the present study, the fertile window of the speakers was determined using LH tests and confirmed with hormone assays of saliva. We minimized potential confounding variables such as irregularity of menstrual cycle, mother tongue, smoking, respiratory diseases, time of the day, and background noise.

Taken together, the present study offers additional evidence that women’s voices subtly change depending on their current menstrual cycle phase and depending on the attractiveness and sex of the stimulus speaker. Importantly, an effect of menstrual cycle was only found when responding to stimulus speakers and not when reading the sentences aloud, suggesting that cycle-dependent voice changes need a social trigger to unfold.

## Data Availability

The original contributions presented in the study are included in the article/Supplementary material, further inquiries can be directed to the corresponding author.
