# Supplementary material for: Voice of a woman: influence of interaction partner characteristics on cycle dependent vocal changes in women
Source: Front Psychol. 2024 Dec 13;15:1401158. doi: 10.3389/fpsyg.2024.1401158 (PMC11671799; doi:10.3389/fpsyg.2024.1401158)
Supplement: Supplementary file 2 [file Data_Sheet_2.PDF]

Supplemetary Material

Table S1

Sentences used

Practice trials	
**German (original)**	**English translation**
Der Autor schreibt das Kapitel.	The author writes the chapter.
Der Fahrer lenkt den Wagen.	The driver steers the car.
Der Hase frisst die Möhre.	The rabbit eats the carrot.

Sentences Block 1 and 2	
**German (original)**	**English translation**
Der Makler erhält den Zuschlag.	The broker is awarded the contract.
Der Postbote bringt den Brief.	The postman brings the letter.
Der Richter verliest das Urteil.	The judge reads out the verdict.
Der Schlüssel öffnet die Tür.	The key opens the door.
Der Zug passiert die Ortschaft.	The train passes the town.
Die Ärztin ruft die Schwester.	The doctor calls the nurse.
Die Biene macht den Honig.	The bee makes the honey.
Die Eltern kaufen das Spielzeug.	The parents buy the toy.
Die Kundin kennt den Laden.	The customer knows the store.
Die Limonade löscht den Durst.	The lemonade quenches the thirst.
Die Nachricht erreicht die Stadt.	The news reaches the city.
Die Postfrau bringt die Zeitung.	The postwoman brings the newspaper.
Die Seife reinigt die Haut.	The soap cleanses the skin.
Das Buch presst das Kleeblatt.	The book presses the cloverleaf.
Das Dienstmädchen deckt den Tisch.	The maid sets the table.
Das Fass enthält den Branntwein.	The barrel contains the brandy.
Das Gold verziert den Rahmen.	The gold decorates the frame.
Das Schaf füttert das Lamm.	The sheep feeds the lamb.
Das Schiff erreicht den Hafen.	The ship reaches the port.
Das Wasser bedeckt den Asphalt.	The water covers the asphalt.
Das Wort verändert die Welt.	The word changes the world.

Note: Written (Block 1) and spoken sentences (Block 2) were identical in content.

Table S2

Interactions in the 2x2x2 ANOVAs

	F	*p*	η_p^2
**F0**			
CyclePhase * SpeakerSex	3.381	.073	.076
CyclePhase * SpeakerVocalAttractiveness	0.002	.968	.000
SpeakerSex * SpeakerVocalAttractiveness	0.203	.655	.005
CyclePhase * SpeakerSex * SpeakerVocalAttractiveness	0.472	.496	.011
**F0 SD**			
CyclePhase * SpeakerSex	2.825	.100	.064
CyclePhase * SpeakerVocalAttractiveness	0.550	.462	.013
SpeakerSex * SpeakerVocalAttractiveness	0.541	.466	.013
CyclePhase * SpeakerSex * SpeakerVocalAttractiveness	0.008	.928	.000
**$F0_{min}$**			
CyclePhase * SpeakerSex	0.405	.528	.010
CyclePhase * SpeakerVocalAttractiveness	0.290	.593	.007
SpeakerSex * SpeakerVocalAttractiveness	2.361	.132	.054
CyclePhase * SpeakerSex * SpeakerVocalAttractiveness	0.209	.650	.005
**$F0_{max}$**			
CyclePhase * SpeakerSex	2.402	.129	.055
CyclePhase * SpeakerVocalAttractiveness	2.330	.135	.054
SpeakerSex * SpeakerVocalAttractiveness	2.835	.100	.065
CyclePhase * SpeakerSex * SpeakerVocalAttractiveness	0.006	.941	.000
**CoG**			
CyclePhase * SpeakerSex	0.008	.930	.000
CyclePhase * SpeakerVocalAttractiveness	0.011	.916	.000
SpeakerSex * SpeakerVocalAttractiveness	1.435	.238	.034
CyclePhase * SpeakerSex * SpeakerVocalAttractiveness	1.173	.285	.028

	F	*p*	η_p^2
**F1**			
CyclePhase * SpeakerSex	0.398	.531	.010
CyclePhase * SpeakerVocalAttractiveness	0.706	.406	.017
SpeakerSex * SpeakerVocalAttractiveness	0.003	.959	.000
CyclePhase * SpeakerSex * SpeakerVocalAttractiveness	0.009	.924	.000
**F2**			
CyclePhase * SpeakerSex	0.001	.974	.000
CyclePhase * SpeakerVocalAttractiveness	0.040	.842	.001
SpeakerSex * SpeakerVocalAttractiveness	0.905	.347	.022
CyclePhase * SpeakerSex * SpeakerVocalAttractiveness	0.094	.760	.002
**F3**			
CyclePhase * SpeakerSex	4.048	.051	.090
CyclePhase * SpeakerVocalAttractiveness	0.005	.943	.000
SpeakerSex * SpeakerVocalAttractiveness	1.554	.220	.037
CyclePhase * SpeakerSex * SpeakerVocalAttractiveness	0.692	.410	.017
**F4**			
CyclePhase * SpeakerSex	0.010	.922	.000
CyclePhase * SpeakerVocalAttractiveness	0.171	.681	.004
SpeakerSex * SpeakerVocalAttractiveness	0.018	.894	.000
CyclePhase * SpeakerSex * SpeakerVocalAttractiveness	0.116	.732	.003
**HNR**			
CyclePhase * SpeakerSex	0.100	.753	.002
CyclePhase * SpeakerVocalAttractiveness	0.112	.740	.003
SpeakerSex * SpeakerVocalAttractiveness	0.958	.334	.023
CyclePhase * SpeakerSex * SpeakerVocalAttractiveness	0.345	.560	.008

	F	*p*	η_p^2
**Jitter**			
CyclePhase * SpeakerSex	0.363	.550	.009
CyclePhase * SpeakerVocalAttractiveness	0.383	.539	.009
SpeakerSex * SpeakerVocalAttractiveness	0.170	.682	.004
CyclePhase * SpeakerSex * SpeakerVocalAttractiveness	0.010	.920	.000
**Shimmer**			
CyclePhase * SpeakerSex	0.490	.488	.012
CyclePhase * SpeakerVocalAttractiveness	0.138	.712	.003
SpeakerSex * SpeakerVocalAttractiveness	0.051	.822	.001
CyclePhase * SpeakerSex * SpeakerVocalAttractiveness	0.000	.995	.000
**IntensSD**			
CyclePhase * SpeakerSex	0.222	.640	.005
CyclePhase * SpeakerVocalAttractiveness	0.152	.699	.004
SpeakerSex * SpeakerVocalAttractiveness	0.392	.535	.009
CyclePhase * SpeakerSex * SpeakerVocalAttractiveness	0.000	.994	.000

Table S3

Phonetic analysis of female stimulus speakers' voices: Attractive versus unattractive voices.

	Female stimulus speakers with attractive voices		Female stimulus speakers with unattractive voices		*T*-tests	
	M	*SD*	*M*	*SD*	*T*(14)	*p*
F0 [Hz]	216.9	11.5	223.5	15.7	–.948	.36
F0 SD [Hz]	42.4	8.3	39.5	6.1	.801	.44
F0 min [Hz]	162.9	6.9	156.6	18.0	.923	.38
F0 max [Hz]	386.1	58.2	364.4	33.5	.914	.38
Centre of Gravity [Hz]	828.9	231.5	593.9	87.7	2.686	**.018**
F1 [Hz]	668.1	29.2	615.7	27.5	3.689	**.002**
F2 [Hz]	1846.5	44.8	1809.2	29.9	1.955	.07
F3 [Hz]	2933.8	58.5	2820.6	60.5	3.805	**.002**
F4 [Hz]	3896.6	54.4	3850.7	61.3	1.584	.14
HNR [dB]	13.3	1.0	14.2	1.2	–1.666	.12
Jitter [%]	.02	.00	.02	.00	–.232	.82
Shimmer [%]	.09	.01	.09	.01	–.277	.79
Intensity SD [dB]	9.9	.6	9.8	.7	.232	.82

Table S4
Phonetic analysis of male stimulus speakers' voices: Attractive versus unattractive voices.

	Male stimulus speakers with attractive voices		Male stimulus speakers with unattractive voices		*T*-tests	
	M	*SD*	*M*	*SD*	*T*(14)	*p*
F0 [Hz]	107.1	8.3	123.5	19.6	–2.188	**.046**
F0 SD [Hz]	17.4	3.6	15.5	3.3	1.117	.28
F0 min [Hz]	80.3	3.0	95.1	12.3	–3.311	**.005**
F0 max [Hz]	155.0	11.7	170.2	25.8	–1.510	.15
Centre of Gravity [Hz]	702.7	165.6	503.0	109.9	2.843	**.013**
F1 [Hz]	650.1	29.8	623.2	61.3	1.119	.28
F2 [Hz]	1769.4	45.0	1704.1	44.6	2.915	**.011**
F3 [Hz]	2780.6	50.0	2701.4	51.2	3.128	**.007**
F4 [Hz]	3747.2	68.6	3662.9	83.0	2.213	**.044**
HNR [dB]	9.6	.5	10.6	2.4	–1.087	.30
Jitter [%]	.03	.00	.03	.01	.185	.86
Shimmer [%]	.11	.01	.12	.02	–.631	.54
Intensity SD [dB]	8.9	.8	9.1	.9	–.376	.71

Table S5

Phonetic analysis of the stimulus speakers' voices, female versus male voices.

	Voices of female stimulus speakers		Voices of male stimulus speakers		*T*-tests	
	M	*SD*	*M*	*SD*	*T*(15)	*p*
F0 [Hz]	217.4	13.3	115.3	16.8	22.84	**< .001**
F0 SD [Hz]	32.3	3.3	16.5	3.5	14.33	**< .001**
F0 min [Hz]	159.2	13.8	87.7	11.6	13.70	**< .001**
F0 max [Hz]	317.5	23.9	162.6	20.9	20.48	**< .001**
Centre of Gravity [Hz]	711.4	208.1	602.9	170.5	1.90	.08
F1 [Hz]	696.3	42.1	636.7	48.6	3.87	**.002**
F2 [Hz]	1956.3	56.0	1736.9	54.6	11.80	**< .001**
F3 [Hz]	3060.7	104.1	2740.5	64.0	9.56	**< .001**
F4 [Hz]	4153.8	90.1	3704.1	85.3	12.72	**< .001**
HNR [dB]	13.8	1.2	10.1	1.7	6.60	**< .001**
Jitter [%]	.023	.004	.033	.006	-5.69	**< .001**
Shimmer [%]	.087	.009	.116	.016	-8.19	**< .001**
Intensity SD [dB]	9.9	.6	9.4	.9	1.84	.09

Table S6
Phonetic analysis of the women's voices in the control condition (reading aloud written sentences), results when predefined frequency range is 100-500 Hz (as suggested for female voices) compared to when predefined frequency range is 75-600 Hz (default settings of Praat software).

	Frequency range in presettings 100-500 Hz		Frequency range in presettings 75-600 Hz		*T*-tests	
	M	*SD*	*M*	*SD*	*T*(41)	*p*
F0 [Hz]	209.6	17.0	211.3	18.0	-3.07	**0.004**
F0 SD [Hz]	33.8	6.2	50.4	10.9	-14.45	**< .001**
F0 min [Hz]	147.8	17.2	132.4	27.5	7.02	**< .001**
F0 max [Hz]	323.8	25.2	398.0	40.4	-17.54	**< .001**
HNR [dB]	13.2	2.1	12.7	1.9	12.37	**< .001**
Jitter [%]	0.0263	0.0055	0.0260	0.0044	1.23	.23
Shimmer [%]	0.0900	0.0124	0.0904	0.0118	-2.92	**0.006**

Table S7
Phonetic analysis of the women's voices in the experimental condition (reproduction of spoken sentences), results when predefined frequency range is 100-500 Hz (as suggested for female voices) compared to when predefined frequency range is 75-600 Hz (default settings of Praat software).

	Frequency range in presettings 100-500 Hz		Frequency range in presettings 75-600 Hz		*T*-tests	
	M	*SD*	*M*	*SD*	*T*(41)	*p*
F0 [Hz]	208.6	17.3	209.5	19.3	-1.33	.19
F0 SD [Hz]	34.7	5.9	52.5	10.0	-17.07	**< .001**
F0 min [Hz]	140.6	17.9	125.4	25.7	7.62	**< .001**
F0 max [Hz]	325.0	26.8	405.1	40.1	-21.05	**< .001**
HNR [dB]	13.1	1.8	12.6	1.6	8.267	**< .001**
Jitter [%]	0.0271	0.0053	0.0263	0.0036	1.963	.056
Shimmer [%]	0.0912	0.0136	0.0913	0.0127	-0.243	0.81

Correlation Matrix: Baseline Condition; Late follicular phase

		T	Cort	P	E	meanF0	F0_SD	F0min	F0max	HNR	jitter	shimmer	F1	F2	F3	F4	CoG
T	Pearson's r	—															
	df	—															
	p-value	—															
Cort	Pearson's r	0.302	—														
	df	40	—														
	p-value	0.052	—														
P	Pearson's r	0.227	0.16	—													
	df	40	40	—													
	p-value	0.148	0.313	—													
E	Pearson's r	-0.231	-0.104	-0.152	—												
	df	40	40	40	—												
	p-value	0.141	0.514	0.335	—												
meanF0	Pearson's r	0.065	0.058	0.3	-0.068	—											
	df	40	40	40	40	—											
	p-value	0.682	0.714	0.053	0.671	—											
F0_SD	Pearson's r	-0.142	0.002	0.057	0.095	-0.006	—										
	df	40	40	40	40	40	—										
	p-value	0.37	0.99	0.718	0.551	0.972	—										
F0min	Pearson's r	0.069	-0.117	-0.032	0.096	0.626 ***	-0.542 ***	—									
	df	40	40	40	40	40	40	—									
	p-value	0.663	0.461	0.841	0.546	< .001	< .001	—									
F0max	Pearson's r	-0.092	-0.228	0.18	0.265	0.47 **	0.511 ***	0.207	—								
	df	40	40	40	40	40	40	40	—								
	p-value	0.564	0.147	0.255	0.09	0.002	< .001	0.189	—								
HNR	Pearson's r	-0.033	-0.196	0.112	0.057	0.469 **	-0.421 **	0.567 ***	0.232	—							
	df	40	40	40	40	40	40	40	40	—							
	p-value	0.835	0.213	0.48	0.718	0.002	0.005	< .001	0.139	—							
jitter	Pearson's r	0.049	0.16	0.013	-0.138	-0.527 ***	0.381 *	-0.516 ***	-0.163	-0.819 ***	—						
	df	40	40	40	40	40	40	40	40	40	—						
	p-value	0.756	0.312	0.937	0.382	< .001	0.013	< .001	0.302	< .001	—						
shimmer	Pearson's r	-0.074	0.195	0.004	-0.04	-0.371 *	0.446 **	-0.416 **	0.022	-0.761 ***	0.899 ***	—					
	df	40	40	40	40	40	40	40	40	40	40	—					
	p-value	0.642	0.215	0.981	0.802	0.016	0.003	0.006	0.892	< .001	< .001	—					
F1	Pearson's r	-0.124	-0.101	-0.189	0.232	-0.295	-0.105	-0.248	-0.233	-0.251	0.096	0	—				
	df	40	40	40	40	40	40	40	40	40	40	40	—				
	p-value	0.434	0.525	0.23	0.139	0.058	0.506	0.114	0.138	0.108	0.546	0.999	—				
F2	Pearson's r	0.204	0.099	0.123	-0.036	-0.203	-0.058	-0.252	-0.307 *	-0.373 *	0.143	0.118	0.526 ***	—			
	df	40	40	40	40	40	40	40	40	40	40	40	40	—			
	p-value	0.195	0.533	0.439	0.819	0.198	0.713	0.108	0.048	0.015	0.367	0.458	< .001	—			
F3	Pearson's r	0.149	-0.076	-0.094	-0.071	-0.272	-0.348 *	-0.059	-0.399 **	-0.085	-0.084	-0.161	0.413 **	0.555 ***	—		
	df	40	40	40	40	40	40	40	40	40	40	40	40	40	—		
	p-value	0.347	0.632	0.555	0.656	0.082	0.024	0.709	0.009	0.591	0.598	0.309	0.007	< .001	—		
F4	Pearson's r	-0.03	-0.099	0.113	-0.126	0.098	-0.234	0.203	-0.144	0.194	-0.424 **	-0.34 *	0.138	0.407 **	0.384 *	—	
	df	40	40	40	40	40	40	40	40	40	40	40	40	40	40	—	
	p-value	0.848	0.532	0.476	0.425	0.537	0.136	0.197	0.362	0.219	0.005	0.028	0.383	0.008	0.012	—	
CoG	Pearson's r	0.027	0.286	-0.114	-0.19	-0.071	0.36 *	-0.245	-0.022	-0.642 ***	0.341 *	0.328 *	0.092	0.268	0.062	0.129	—
	df	40	40	40	40	40	40	40	40	40	40	40	40	40	40	40	—
	p-value	0.863	0.066	0.473	0.228	0.657	0.019	0.118	0.889	< .001	0.027	0.034	0.562	0.086	0.695	0.416	—

Note. * p < .05, ** p < .01, *** p < .001

Correlation Matrix: Baseline Condition; Luteal phase

		T	Cort	P	E	meanF0	F0_SD	F0min	F0max	HNR	jitter	shimmer	F1	F2	F3	F4	CoG
T	Pearson's r	—															
	df	—															
	p-value	—															
Cort	Pearson's r	0.515 ***	—														
	df	40	—														
	p-value	< .001	—														
P	Pearson's r	-0.087	0.127	—													
	df	40	40	—													
	p-value	0.585	0.424	—													
E	Pearson's r	0.13	-0.129	-0.102	—												
	df	40	40	40	—												
	p-value	0.413	0.414	0.522	—												
meanF0	Pearson's r	-0.153	-0.164	-0.003	-0.329 *	—											
	df	40	40	40	40	—											
	p-value	0.333	0.3	0.984	0.034	—											
F0_SD	Pearson's r	0.184	-0.071	0.013	-0.018	0.171	—										
	df	40	40	40	40	40	—										
	p-value	0.243	0.655	0.936	0.909	0.28	—										
F0min	Pearson's r	-0.123	-0.163	-0.097	-0.161	0.62 ***	-0.488 **	—									
	df	40	40	40	40	40	40	—									
	p-value	0.437	0.302	0.542	0.308	< .001	0.001	—									
F0max	Pearson's r	0.163	0.027	0.067	-0.154	0.513 ***	0.807 ***	-0.094	—								
	df	40	40	40	40	40	40	40	—								
	p-value	0.303	0.865	0.674	0.332	< .001	< .001	0.556	—								
HNR	Pearson's r	-0.186	0.021	0.288	-0.328 *	0.494 ***	-0.293	0.509 ***	0.072	—							
	df	40	40	40	40	40	40	40	40	—							
	p-value	0.237	0.893	0.064	0.034	< .001	0.06	< .001	0.65	—							
jitter	Pearson's r	0.199	0.107	-0.226	0.327 *	-0.518 ***	0.231	-0.484 **	-0.05	-0.889 ***	—						
	df	40	40	40	40	40	40	40	40	40	—						
	p-value	0.207	0.5	0.15	0.035	< .001	0.141	0.001	0.755	< .001	—						
shimmer	Pearson's r	0.116	0.007	-0.199	0.333 *	-0.433 **	0.214	-0.44 **	-0.02	-0.823 ***	0.908 ***	—					
	df	40	40	40	40	40	40	40	40	40	40	—					
	p-value	0.463	0.967	0.207	0.031	0.004	0.173	0.004	0.9	< .001	< .001	—					
F1	Pearson's r	-0.241	-0.253	-0.121	0.176	-0.154	0.198	-0.19	0.022	-0.188	0.109	0.09	—				
	df	40	40	40	40	40	40	40	40	40	40	40	—				
	p-value	0.124	0.105	0.445	0.266	0.33	0.208	0.228	0.892	0.233	0.492	0.571	—				
F2	Pearson's r	-0.301	-0.229	-0.213	0.147	-0.257	0.062	-0.365 *	-0.151	-0.295	0.151	0.207	0.487 **	—			
	df	40	40	40	40	40	40	40	40	40	40	40	40	—			
	p-value	0.053	0.145	0.175	0.353	0.1	0.698	0.018	0.339	0.058	0.338	0.189	0.001	—			
F3	Pearson's r	-0.262	-0.122	-0.216	0.126	-0.205	-0.215	0.019	-0.286	0.03	-0.092	-0.078	0.373 *	0.563 ***	—		
	df	40	40	40	40	40	40	40	40	40	40	40	40	40	—		
	p-value	0.093	0.442	0.17	0.427	0.192	0.171	0.904	0.066	0.85	0.562	0.621	0.015	< .001	—		
F4	Pearson's r	-0.303	-0.259	0.135	0.063	0.013	0.046	-0.002	-0.053	0.283	-0.341 *	-0.303	0.304	0.406 **	0.435 **	—	
	df	40	40	40	40	40	40	40	40	40	40	40	40	40	40	—	
	p-value	0.051	0.098	0.394	0.69	0.935	0.771	0.992	0.737	0.07	0.027	0.051	0.05	0.008	0.004	—	
CoG	Pearson's r	-0.046	-0.202	-0.217	0.07	0.006	0.395 **	-0.202	0.168	-0.467 **	0.223	0.206	0.017	0.265	0.025	0.077	—
	df	40	40	40	40	40	40	40	40	40	40	40	40	40	40	40	—
	p-value	0.771	0.2	0.167	0.659	0.969	0.01	0.199	0.287	0.002	0.155	0.19	0.913	0.09	0.874	0.63	—

Note. * p < .05, ** p < .01, *** p < .001

Correlation Matrix: Treatment Condition; Late follicular phase

Correlation Matrix

		T	Cort	P	E	meanF0	F0_SD	F0min	F0max	HNR	jitter	shimmer	F1	F2	F3	F4	CoG
T	Pearson's r	—															
	df	—															
	p-value	—															
Cort	Pearson's r	0.302	—														
	df	40	—														
	p-value	0.052	—														
P	Pearson's r	0.227	0.16	—													
	df	40	40	—													
	p-value	0.148	0.313	—													
E	Pearson's r	-0.231	-0.104	-0.152	—												
	df	40	40	40	—												
	p-value	0.141	0.514	0.335	—												
meanF0	Pearson's r	0.054	0.14	0.337 *	0.018	—											
	df	40	40	40	40	—											
	p-value	0.734	0.375	0.029	0.91	—											
F0_SD	Pearson's r	-0.032	0.125	0.199	0.019	0.195	—										
	df	40	40	40	40	40	—										
	p-value	0.842	0.429	0.206	0.906	0.215	—										
F0min	Pearson's r	0.012	-0.013	0.029	0.147	0.538 ***	-0.545 ***	—									
	df	40	40	40	40	40	40	—									
	p-value	0.94	0.935	0.855	0.355	< .001	< .001	—									
F0max	Pearson's r	0.082	0.092	0.174	0.171	0.614 ***	0.7 ***	0.07	—								
	df	40	40	40	40	40	40	40	—								
	p-value	0.607	0.563	0.271	0.279	< .001	< .001	0.659	—								
HNR	Pearson's r	-0.077	-0.21	0.041	0.184	0.246	-0.279	0.468 **	0.079	—							
	df	40	40	40	40	40	40	40	40	—							
	p-value	0.628	0.182	0.797	0.244	0.117	0.073	0.002	0.62	—							
jitter	Pearson's r	-0.094	0.174	0.071	-0.217	-0.41 **	0.242	-0.477 **	-0.117	-0.717 ***	—						
	df	40	40	40	40	40	40	40	40	40	—						
	p-value	0.555	0.27	0.654	0.168	0.007	0.123	0.001	0.461	< .001	—						
shimmer	Pearson's r	-0.303	0.096	0.147	-0.097	-0.105	0.297	-0.288	0.026	-0.51 ***	0.759 ***	—					
	df	40	40	40	40	40	40	40	40	40	40	—					
	p-value	0.051	0.545	0.353	0.541	0.507	0.056	0.064	0.872	< .001	< .001	—					
F1	Pearson's r	0.126	-0.074	-0.176	0.127	-0.319 *	-0.155	-0.159	-0.209	-0.214	0.15	-0.065	—				
	df	40	40	40	40	40	40	40	40	40	40	40	—				
	p-value	0.428	0.642	0.265	0.424	0.04	0.326	0.313	0.185	0.174	0.344	0.683	—				
F2	Pearson's r	0.212	0.169	0.221	-0.083	-0.092	0.069	-0.203	-0.085	-0.288	0.061	0.042	0.465 **	—			
	df	40	40	40	40	40	40	40	40	40	40	40	40	—			
	p-value	0.178	0.286	0.159	0.602	0.56	0.664	0.196	0.593	0.065	0.703	0.792	0.002	—			
F3	Pearson's r	0.227	-0.116	-0.072	0.013	-0.234	-0.362 *	0.014	-0.357 *	0	-0.212	-0.369 *	0.523 ***	0.552 ***	—		
	df	40	40	40	40	40	40	40	40	40	40	40	40	40	—		
	p-value	0.149	0.464	0.652	0.933	0.136	0.019	0.929	0.02	0.998	0.177	0.016	< .001	< .001	—		
F4	Pearson's r	-0.048	-0.088	0.152	-0.031	0.158	-0.125	0.112	-0.143	0.186	-0.429 **	-0.164	0.278	0.457 **	0.431 **	—	
	df	40	40	40	40	40	40	40	40	40	40	40	40	40	40	—	
	p-value	0.764	0.58	0.335	0.848	0.318	0.431	0.479	0.367	0.238	0.005	0.298	0.074	0.002	0.004	—	
CoG	Pearson's r	0.1	0.281	-0.121	-0.151	0.038	0.281	-0.229	0.095	-0.506 ***	0.078	-0.016	0.117	0.263	0.11	0.186	—
	df	40	40	40	40	40	40	40	40	40	40	40	40	40	40	40	—
	p-value	0.529	0.072	0.444	0.34	0.811	0.071	0.144	0.55	< .001	0.624	0.922	0.459	0.092	0.488	0.239	—

Note. * p < .05, ** p < .01, *** p < .001

Correlation Matrix: Treatment Condition; Luteal phase

Correlation Matrix

		T	Cort	P	E	meanF0	F0_SD	F0min	F0max	HNR	jitter	shimmer	F1	F2	F3	F4	CoG
T	Pearson's r	—															
	df	—															
	p-value	—															
Cort	Pearson's r	0.515 ***	—														
	df	40	—														
	p-value	< .001	—														
P	Pearson's r	-0.087	0.127	—													
	df	40	40	—													
	p-value	0.585	0.424	—													
E	Pearson's r	0.13	-0.129	-0.102	—												
	df	40	40	40	—												
	p-value	0.413	0.414	0.522	—												
meanF0	Pearson's r	-0.073	-0.183	-0.049	-0.195	—											
	df	40	40	40	40	—											
	p-value	0.647	0.245	0.758	0.216	—											
F0_SD	Pearson's r	0.162	0.021	0.003	-0.064	0.219	—										
	df	40	40	40	40	40	—										
	p-value	0.306	0.896	0.986	0.685	0.163	—										
F0min	Pearson's r	-0.176	-0.371 *	-0.106	-0.02	0.645 ***	-0.415 **	—									
	df	40	40	40	40	40	40	—									
	p-value	0.266	0.016	0.505	0.9	< .001	0.006	—									
F0max	Pearson's r	0.171	-0.12	0.017	-0.14	0.612 ***	0.762 ***	0.147	—								
	df	40	40	40	40	40	40	40	—								
	p-value	0.278	0.451	0.914	0.378	< .001	< .001	0.353	—								
HNR	Pearson's r	-0.1	0.018	0.233	-0.295	0.424 **	-0.206	0.5 ***	0.207	—							
	df	40	40	40	40	40	40	40	40	—							
	p-value	0.531	0.912	0.137	0.058	0.005	0.19	< .001	0.189	—							
jitter	Pearson's r	0.055	0.066	-0.129	0.31 *	-0.625 ***	0.112	-0.604 ***	-0.322 *	-0.81 ***	—						
	df	40	40	40	40	40	40	40	40	40	—						
	p-value	0.731	0.677	0.414	0.046	< .001	0.48	< .001	0.037	< .001	—						
shimmer	Pearson's r	-0.093	-0.003	-0.036	0.304	-0.44 **	0.227	-0.527 ***	-0.145	-0.656 ***	0.891 ***	—					
	df	40	40	40	40	40	40	40	40	40	40	—					
	p-value	0.56	0.986	0.821	0.05	0.004	0.148	< .001	0.359	< .001	< .001	—					
F1	Pearson's r	-0.391 *	-0.342 *	-0.18	0.274	-0.234	0.008	-0.16	-0.199	-0.363 *	0.384 *	0.441 **	—				
	df	40	40	40	40	40	40	40	40	40	40	40	—				
	p-value	0.01	0.027	0.255	0.08	0.136	0.959	0.31	0.207	0.018	0.012	0.003	—				
F2	Pearson's r	-0.403 **	-0.195	-0.151	0.054	-0.131	0.067	-0.226	-0.13	-0.266	0.235	0.308 *	0.562 ***	—			
	df	40	40	40	40	40	40	40	40	40	40	40	40	—			
	p-value	0.008	0.215	0.34	0.733	0.408	0.672	0.151	0.413	0.089	0.133	0.048	< .001	—			
F3	Pearson's r	-0.363 *	-0.237	-0.241	0.061	-0.16	-0.23	0.054	-0.229	0.103	-0.058	-0.049	0.464 **	0.612 ***	—		
	df	40	40	40	40	40	40	40	40	40	40	40	40	40	—		
	p-value	0.018	0.13	0.124	0.699	0.311	0.143	0.734	0.144	0.517	0.715	0.756	0.002	< .001	—		
F4	Pearson's r	-0.361 *	-0.26	0.078	-0.044	0.152	-0.105	0.213	0.045	0.248	-0.354 *	-0.251	0.345 *	0.439 **	0.439 **	—	
	df	40	40	40	40	40	40	40	40	40	40	40	40	40	40	—	
	p-value	0.019	0.096	0.622	0.784	0.335	0.509	0.176	0.779	0.113	0.021	0.11	0.025	0.004	0.004	—	
CoG	Pearson's r	-0.03	-0.226	-0.222	0.13	0.082	0.283	-0.022	0.19	-0.53 ***	0.126	0.057	0.16	0.217	0.007	0.201	—
	df	40	40	40	40	40	40	40	40	40	40	40	40	40	40	40	—
	p-value	0.849	0.15	0.157	0.411	0.606	0.07	0.891	0.227	< .001	0.425	0.721	0.313	0.168	0.964	0.202	—

Note. * p < .05, ** p < .01, *** p < .001
